# Charity Hospital Clinical Reports

**Published:** 1893-02

**Authors:** Robert Morris

**Affiliations:** Resident Student; New Orleans


### CHARITY HOSPITAL CLINICAL REPORTS.

BY ROBERT MORRIS, RESIDENT STUDENT.

### Acute Mercurialization and Abscess of the Liver.*

GENTLMEN.—The history of this man which antedates the symptoms of hepatic abscess, relates to an accident, even at this day too common in the practice of medicine. Twenty-three days ago the patient feeling ill and suffering of constipation and vomiting, sought medical relief and received a prescription containing calomel. In the course of twenty-fonr hours he began to show the symptoms of acute mercurialization. These symptoms became greatly intensified and during the past three weeks he has suffered seriously of the local phenomena and the constitutional symptoms incident to the over-action of the mercurial. At this time his gums are congested and eroded; the teeth are loosened in their sockets and covered with sordes, the flow of saliva is increased and the breath exceeding fetid.

We are impressed by the pallor of this patient as presented in the skin and mucous membranes. Observe how pale and anæmic, in appearance, are his lips and the mucous surfaces of the tongue and buccal cavity. In acute mercurialization we expect such evidences of blood dyscrasia as you here observe for mercurials in excessive doses, or in the peculiarly susceptible, are agents very destructive to red blood corpuscles.

In the administration of the mercurials there are cautions to be observed which I desire to impress with all possible emphasis. In any state of constipation, where you have reason to doubt the speedy action of calomel, select some purgative attended with less risks. During the alterative treatment with the mercurials, we may, in exigencies, which we have discussed elsewhere, keep the patient, for a time, on the border ground between the full effect of therapeutic doses and the symptoms of toxic over action. But the observance of certain cautions will usually avert mercurial accidents. We are warned by the hyperæmia of the gums, the tenderness of the teeth, the increased salivary flow, and the mercurial fetor of the breath. All these symptoms may be detected in advance of that state of exaggerated phenomena, such as characterizes the accident which occurred in the case before you.

*Abstract of a Clinical Lecture by Prof. A. B. Wilson, M. D.

There are certain classes of patients who are easily affected by the mercurials, and unless proper cautions be observed, the action of the agent may unexpectedly exceed the therapeutic effect. Here I desire to impart the statement that the therapeutic effect of any one of the preparations of mercury is secured short of the symptoms of acute mercurialization. Mercurial salivation is one of the most perilous accidents that can happen in the course of a serious illness; and one that always makes the convalescence tedious and precarious.

Be cautious in the administration of calomel in the constipation of the anæmic. Alterative doses of the mercurial easily overact in the strumous and in cases of depression, and in the low forms of chronic fevers, when the diminished blood pressure in all organs concerned in eliminating the mercurial favors a cumulative action. In renal and structural hepatic diseases the mercurials easily overact. Cases of chronic nephritis are easily salivated. Be exceedingly cautious in the administration of mercurials in the treatment of such cases of disease.

The treatment in the case before us, for the relief of the distressing accident which has occurred, comprises lotions for the relief of local symptoms, the internal administration of an agent to promote elimination of the mercurial, aud the assistance of some chalybeate to repair the destructive effects on the red blood corpuscles. For local use, solutions of boric acid, chlorate of potassium and the tincture of myrrh, are perhaps the most servicable. The syrup of the iodide of iron, the purpose of which you now comprehend, is perhaps one of the best of all agents we can employ to meet the general therapeutic indications.

So much for the accident that has attracted our attention before we come to the discussion of the present disease—an abscess of the left lobe of the liver.

The most attractive local symptoms is the intense pain over the hepatic region, the point of maximum intensity being over the area of swelling you see. Even the respiratory movements are painful and pressure is unbearable. The upper boundary line of the liver is normal, the anterior border of the right lobe exceeds but very little the natural limits. The left lobe of the organ is enlarged, its border depressed three or four fingers breadth below its usual position, and the entire lobe bulging and painful. Then the hepatic symptoms are localized. These, in connection with the history of fever, and occurring in an acute disease, make the diagnosis almost positive. The aspiration which we now perform, makes the diagnosis conclusive.

What course of treatment shall we now adopt? We must be prudent. The diagnosis of an abscess of the liver does not always redound to the benefit of the patient. The critical condition of this patient will not admit of any heroic procedure. The exigency is not increased by high temperature, and, therefore, surgical treatment, at this time, is not the essential indication. Moreover, we do not know that adhesions have occurred between the convexity of the liver and the abdominal wall, and when in doubt, an incision into an hepatic abscess is attended with criminal risk. We do not observe any redness of the surface over this swelling, nor œdema, nor superficial fluctuation, nor indeed, any evidence of adhesive fixation; and therefore, we deem it prudent to aspirate this abscess. By this procedure we have withdrawn six ounces of pus, and by reducing the distension, relieved pain. While making the patient comfortable, we gain time and prepare him for any surgical operation that may in the future become necessary.

We propose to aspirate this abscess once or twice a week, puncturing always in the same location. In this way we excite adhesive inflammation, and later, should the necessity arise, we will either insert, by the same tract, a trocar of large size, in order to use the canula as a permanent drain, or we will make an incision through the area of local adhesions. Such an incision should be made cautiously by dissecting down to the peritoneum. If adhesions have not taken place, we are warned, for the time, against further attempt. A short time since, while making an incision as described, I discovered a healthy peritoneal membrane over the region of the abscesss A granule of chloride of zink was retained in the wound, and, after the lapse of twenty-four hours, the abscess was opened with safety.

We should never have a definite and unalterable plan for the treatment of abscess of the liver. Modes of treatment must be adopted according to the special indication of individual cases. Aspiration is the simplest of all kinds of treatment, and applicable in a large number of cases. Abscesses deeply situated in the liver are often most safely treated in this way. Many small abscesses are cured by aspiration, and this is true especially in the cases of children. When the septic symptoms are not urgent aspiration may be attempted in many cases as a safe and simple mode of treatment, and one giving a fair chance of recovery. In any case like the one before us aspiration gives immediate relief of the most distressing symptom, and often increase the chances of recovery by preparing and fortifying patients for necessary surgical operations.

While urging you to be prudent in the management of these cases of hepatic abscess, we trust you will ever be prepared to act promptly and operate decisively in those cases necessitating other modes of surgical treatment. When the pus has escaped the confines of the liver and invaded the thoracic or abdominal walls, treatment by free incision is the approved plan. When aspiration fails to give relief in those cases, where the evidences of adhesions are unmistakable, incise freely, irrigate and properly drain the abscess. In cases where the life of the patient is in jeopardy from pyæmia, and when his strength is being rapidly wasted by high temperature, then we are warranted in making heroic efforts to reach and evacuate the pus. Some caustics, as the chloride of zinc, retained in an incision over the serous membrane, will soon prepare the way for an easy entrance into the abscess cavity by the scalpel or large trocar and canula. In a case which I now recall, resulting most satisfactorily, a Spencer Wells canula was employed.

What is the natural history of these hepatic abscesses?

They may remain in the organ and gradually destroy tissue to an extent incompatible with life. They may destroy by sepsis. They may escape from the liver and invade the neighboring tissues and organs. The pus may find its way into the thoracic or abdominal parieties. This condition, as we have described, invites and facilitates surgical treatment. The pus may possibly enter one of the neighboring serous cavities. It is much more frequently invited into some tube with contents in motion, the bronchial tubes or the alimentary canal. The most favorable mode of escape is by way of the alimentary canal.

We should not remain inactive and trust to nature's power of healing in the management of cases of abscess of the liver. In the case before us the necessity for action is all the more urgent. The left lobe of the liver is more remotety removed from the safest avenues of escape for the pus of an abscess within its confines. In this instance, if the pus should not invade the abdominal wall, it may escape into the stomach or in the pericardium. In the first instance, the result would be disastrous; in the second, certainly fatal.

It is our purpose, in the management of the case, to aspirate the abscess once or twice a week and continue this mode of treatment as long as the patient improves. Should the case demand more decisive measures, we shall prepare to insert a large trocar-canula, or treat surgically, by incision, antiseptic irrigation and drainage, as the emergency may demand.

